# Australian news media framing of medical tourism in low- and middle-income countries: a content review

**DOI:** 10.1186/1471-2458-13-109

**Published:** 2013-02-05

**Authors:** Michelle Imison, Stephen Schweinsberg

**Affiliations:** 1 Sydney School of Public Health, University of Sydney, Sydney, NSW, Australia; 2 Events, Leisure, Sport, Tourism and Arts, Management Discipline Group, University of Technology, Sydney, Sydney, NSW, Australia

**Keywords:** Australia, Content analysis, Medical tourism, News media, Newspaper, Television, Low– and middle–income countries

## Abstract

**Background:**

Medical tourism – travel across international borders for health care – appears to be growing globally, with patients from high-income nations increasingly visiting low- and middle-income countries to access such services. This paper analyses Australian television and newspaper news and current affairs coverage to examine how medical tourism and these destinations for the practice are represented to media audiences.

**Methods:**

Electronic copies of Australian television (*n* = 66) and newspaper (*n* = 65) items from 2005–2011 about medical care overseas were coded for patterns of reporting (year, format and type) and story characteristics (geographic and medical foci in the coverage, news actors featured and appeals, credibility and risks of the practice mentioned).

**Results:**

Australian media coverage of medical tourism was largely focused on Asia, featuring cosmetic surgery procedures and therapies unavailable domestically. Experts were the most frequently-appearing news actors, followed by patients. Common among the types of appeals mentioned were access to services and low cost. Factors lending credibility included personal testimony, while uncertainty and ethical dilemmas featured strongly among potential risks mentioned from medical tourism.

**Conclusions:**

The Australian media coverage of medical tourism was characterised by a narrow range of medical, geographic and ethical concerns, a focus on individual Australian patients and on content presented as being personally relevant for domestic audiences. Medical tourism was portrayed as an exercise of economically-rational consumer choice, but with no attention given to its consequences for the commodification of health or broader political, medical and ethical implications. In this picture, LMICs were no longer passive recipients of aid but providers of a beneficial service to Australian patients.

## Background

The mainstream news media are central to the formation of public ideas about health and medicine in high-income countries, and about the world beyond our nations’ borders [1,2]. Both broadcast and print coverage in high-income nations tend to provide limited menus of topics and approaches to different areas of news interest, driven by what is logistically and culturally accessible for media outlets, and perceptions of what is personally and strategically important to audiences and governments [3-7]. Previous research that combines a focus on health/medical and foreign news has shed light on the Australian media’s portrayal of health in low- and middle-income countries (LMICs), and demonstrated how little is known about similar coverage of LMICs in other national contexts [8]. In relation to the content of Australian LMIC health coverage, much reporting is simplified – to what might be termed a ‘disease, disaster and despair’ focus – and the imperative of highlighting some Australian domestic element remains especially important [9].

The Australian media reverses some of these expectations and patterns in its coverage of medical tourism, making this phenomenon a particularly interesting one to examine. ‘Medical tourism’ is defined here as individuals – specifically those from high-income nations and often with some intention to include a holiday with their travel – crossing national borders to access non-emergency medical services not otherwise available in their home (source) country because of high costs, long waiting lists, limited health-care capacity or regulatory restrictions [10-12]. Health-related travel has been a characteristic of global tourism since antiquity [13]. However, it is only relatively recently that travelling for medical services has become a distinct practice, for reasons broadly related to the global rise of the middle class, the increased availability of low-cost air travel and developments in medical technology [14]. As a high-income nation, Australia is a prospective source country for medical tourists.

The narrative accounts of Canadian medical tourists indicate that cost was a significant factor among a heterogenous set of motivations that propelled them to seek care overseas [15]. Because of the importance of this consideration in medical-tourism decision-making, much of the recent growth in medical tourism has been in LMICs as a result of their lower costs for labour and construction, preferential tax regimes and cheaper or non-existent practitioner insurance [13,16]. Many countries across Central and South America, eastern Europe and Asia now provide medical tourism services, specialising in particular types of surgery or travel experiences [17,18]. This has numerous, potentially positive consequences for destination countries, including the ability to earn foreign income, the opportunity to raise the standard of domestic health-care by helping to underwrite the expansion of public service-provision and improving coverage by enticing emigrant medical practitioners to return [10,13,14,16]. Although widely-cited figures estimate that medical tourism to Asia will generate US$4.4b in annual revenue for the region by 2012 [19], there is a dearth of reliable information on the numbers of medical tourists and the economic benefit they provide. Even though the phenomenon appears to be growing globally, there are no robust data for any destination country [20,21] and analysis of medical tourist numbers, narrowly defined, would seem to indicate that industry estimates are usually overstated [22]. In addition, there are also major possible downsides for those nations that pursue medical tourism: the failure of financial and medical-personnel gains to ‘trickle down’ to advantage the wider population, increased drift of healthcare workers to particular geographic locations and specialties and the chance that contagions and drug-resistant infections may more easily spread across the globe [23-26].

The growth of medical tourism has been assisted by the development of both travel and medical services in LMICs [27], and their ability to attract international medical tourists relies on the promotion of an image that stresses the quality of available health-care. Exemplary among such services are Bumrungrad Hospital in Bangkok and India’s Apollo Hospitals Group, corporate medical outfits that not only offer treatment in their own facilities for international patients but have also begun acquiring and managing hospitals elsewhere in Asia [23,24]. This state of affairs challenges the usual media depiction in high-income nations of LMICs as inherently ‘unhealthy’ and medically-unsophisticated environments [8]. The limited existing research into the coverage of medical tourism in the English-language media of both destination and source countries has demonstrated that voices of ethical concern have been overwhelmed by medical tourism’s dominant market and consumer discourses [28]. Although a good deal of academic literature on medical tourism refers to media coverage of the phenomenon as a proxy for public interest, most does not look in-depth at media content [16,17,19,29]. This research aims to bridge that gap, reporting on coverage from a large Australian television and newspaper dataset; media-related work to date has focused on Europe and Canada [28]. The purposes of this paper, then, are to analyse the content of relevant television and newspaper items, examine how medical tourism and the LMIC destination countries^a^ for this practice are presented to Australians in their news and current affairs, and explore the possible implications of this portrayal.

## Methods

Television items were drawn from the University of Sydney’s Australian Health News Research Collaboration (AHNRC) digital database. The AHNRC dataset includes all health-related news and current affairs items aired on Sydney’s five free-to-air television stations (three commercial and two at least partly publicly-funded). The sample extends from May 2005, when the database was established, until the end of June 2011, when analysis commenced. The AHNRC’s content and inclusion criteria have been described elsewhere [30]. This television dataset comprised all items that mentioned elective medical care overseas, including items about procedures such as overseas surrogacy and living-donor organ transplants (‘transplant tourism’) whose definition as ‘medical tourism’ might fall outside classifications used elsewhere in the literature [12,31,32]. That these practices are controversial and either heavily regulated or banned in Australia, yet such stories were still broadcast, indicates that there was deemed to be domestic interest in these topics and thus they formed a legitimate part of our dataset.

In order to examine the fullest possible picture of what Australian audiences were shown about medical tourism, these television data were supplemented with print items extracted from the Factiva database of Australian newspaper coverage for the same time-period. We used the search terms ‘medical tourism’, ‘cosmetic tourism’, ‘scalpel tourism’, ‘reproductive tourism’ and ‘transplant tourism’ to locate English-language content that appeared in any non-specialist, non-trade Australian metropolitan or regional newspapers. Excluded were duplicate items, those that made only passing reference to the phenomenon and those concerning inbound medical tourism, since these items invariably focused on what the Australian health system could offer potential patients. Previous research has demonstrated that online news is largely sourced from a small number of existing, traditional news outlets [33]. As television and newspapers therefore offer widely-disseminated content which is also supplied to online outlets, web news was not included in the present study.

The selected television and newspaper content was initially coded in relation to patterns of reporting: year and location of broadcast/publication, format and story type [34] (Table 1). Items were classed as being either ‘news’ or ‘feature’, with content included in the ‘news’ category if there was a discrete trigger for medical tourism having become news – for instance, a political announcement, public event or report of research findings. The ‘feature’ category included media items that were less dependent on such time-bound prompts for their broadcast or publication; they often included strong human-interest elements or reported on medical tourism broadly as a social phenomenon. News and feature items were then further classified as being either focused on or simply mentioning medical tourism. One item of ‘advice’ – a travel journalist’s response to a reader question about medical tourism – and a letter to the editor were also coded.

**Table 1:** Patterns of reporting in Australian newspaper and television coverage of medical tourism, May 2005 – June 2011

**Television stories (*n *= 66)**			
**Year**	**Network type**	**Programme type**	**Story type**
*2005* – 6	*Commercial* – 44	*Current affairs* – 25	*News (main focus)* – 7
*2006* – 3	*Publicly-funded* – 22	*News* – 24	*News (mention)* – 0
*2007* – 22		*Magazine* – 14	*Feature (main focus)* – 45
*2008* – 10		*Discussion* – 3	*Feature (mention)* – 14
*2009* – 6			
*2010* – 17			
*2011* – 2			
**Newspaper stories (*n *= 65)**			
**Year**	**Publication type**	**Source location**	**Story type**
*2005* – 6	*Metropolitan weekday* – 34	*New South Wales* – 26	*News (main focus)* – 22
*2006* – 5	*Metropolitan Sunday* – 21	*Queensland* – 16	*News (mention)* – 3
*2007* – 14	*Community* – 4	*Western Australia* – 9	*Feature (main focus)* – 30
*2008* – 12	*National* – 4	*National* – 4	*Feature (mention)* – 8
*2009* – 6	*Regional weekday* – 2	*Victoria* – 4	*Advice* – 1
*2010* – 14		*Australian Capital Territory* – 3	*Letter to the Editor* – 1
*2011* – 8		*Tasmania* – 2	
		*South Australia* – 1	

We then examined the characteristics of the media coverage by way of a content analysis. The nations, main medical procedures and conditions/treatments mentioned, and any news actors quoted directly (by both type and number) were noted for each television and newspaper item (Tables 2 and 3). A modified version of an existing framework, developed to assess medical tourism websites [35], was then applied to the television and newspaper items. This framework was expanded iteratively by the first author as part of a process of reviewing the coverage and noting important concepts that emerged, while excluding elements of the existing coding schema that were irrelevant to the examination of television and newspaper items – for example, aspects of interactivity. No coding software was used. The broad categories employed were:

• *appeals* – features of medical tourism mentioned in an item, either by the journalist or by a news actor, as attractive for a potential or actual patient,

• *credibility* – dimensions of the medical tourism experience mentioned, either by the journalist or by a news actor, or referenced visually to give it integrity or authority in the mind of a potential or actual patient [16] and

• *risks* – aspects of medical tourism mentioned, either by the journalist or by a news actor, as a source of actual or perceived risk, and perhaps as a reason not to proceed with an overseas medical procedure.

**Table 2:** Characteristics of Australian television coverage of medical tourism, May 2005 – June 2011 (n = 66)*

**National focus ( *n *)**	**Medical focus ( *n *)**	**News actors ( *n *)**	Appeals (%)**	**Credibility (%)**	**Risks (%)**
*India* – 26	*Cosmetic surgery* – 20	*Patient* – 69 (F: 58; Au: 63)	*Access to services (except for reasons of cost)* – 62.1	*Use of personal testimonials* – 50.0	*Ethical dilemmas* – 53.0
*Malaysia* – 12	*Stem-cell therapy* – 15		*Low cost* – 36.4	*Reference to number of international patients* – 42.4	*Uncertainty as to what is on offer* – 50.0
*Thailand* – 10	*Reproductive therapies* – 13	*Expert –* 68 (Au: 36)	*Ability to ‘feel good’* – 21.2	*Logo/branding symbol −*19.7	*Complications* – 34.8
*The Philippines* – 6	*Transplant surgery* – 9	*Medical tourism facilitator/representative of overseas hospital* – 26 (Au: 2)	*Access to ‘medical breakthrough’* – 21.2	*Mention of international accreditation* – 12.1	*Procedural risk* – 22.7
*China* – 5	*Reconstructive surgery* – 4	*Family member/carer* – 22 (F: 6)	*Travel opportunity −*16.7	*Surgeon/practitioner biography or education* – 10.6	*Post-operative care* – 12.1
*Pakistan and Russia* – 2 each	*Orthopaedic surgery* – 3	*Kidney donor* – 13 (F: 3)	*High-quality services* – 13.6	*Ease of contacting practitioner post-procedure* – 6.0	*Lack of legal recourse* – 10.6
*‘Asia’ in general* – 2	*Novel, drug-resistant infection* – 2	*Surrogacy client* – 10 (F: 5; Au: 8)	*No waiting time* – 13.6		*Exposure to novel risks* – 9.1
*No country named* – 3	*Other* – 3	*Other* – 7 (Au: 5)	*State-of-the-art facilities* – 12.1		*Difficulty in contacting practitioner post-procedure* – 4.5
		*‘Vox pop’/audience member* – 6 (Au: 6)	*Surgeon/practitioner expertise* – 10.6		
		*Government spokesperson or official* – 4 (Au: 0)	*Personalised service* – 10.6		
		*Politician* – 4 (Au: 2)	*Access to latest technology* – 4.5		
		*Surrogate mother* – 3 (Au: 1)	*Greater convenience* – 1.5		

* Counts sum to more than 66, as some items cover more than one medical focus, or include more than one news actor.

* * This refers to the percentage of stories to feature mention of the particular appeal, credibility or risk in question.

**Table 3:** Characteristics of Australian newspaper coverage of medical tourism, May 2005 – June 2011 (n = 65)*

**National focus ( *n *)**	**Medical focus ( *n *)**	**News actor ( *n *)**	Appeals (%)**	**Credibility (%)**	**Risks (%)**
*Thailand* – 27	*Cosmetic surgery* – 29	*Expert* – 56 (Au: 45)	*Low cost* – 53.8	*Reference to number of international patients* – 35.4	*Uncertainty as to what is on offer* – 69.2
*India –* 19	*Transplant surgery* – 22	*Patient* – 27 (F: 19; Au: 26)	*Access to services (except for reasons of cost)* – 36.9	*Use of personal testimonials* – 18.5	*Complications* – 47.7
*China* – 12	*Orthopaedic surgery* – 11	*Medical tourism facilitator/representative of overseas hospital* – 23 (Au: 21)	*No waiting time* – 33.8	*Logo/branding symbol* – 15.4	*Ethical dilemmas* – 36.9
*Malaysia and The Philippines* – 11 each	*Dental surgery* – 9	*Government spokesperson or official* – 15 (Au: 6)	*Travel opportunity* – 32.3	*Surgeon/practitioner biography or education* – 12.3	*Exposure to novel risks* – 24.6
*Other* – 8	*Cardiac surgery* – 7	*Family member/carer* – 7 (F: 4)	*Ability to ‘feel good’* – 29.2	*Mention of international accreditation* – 4.6	*Lack of legal recourse* – 21.5
*Pakistan* – 6	*Reproductive therapies* – 5	*Other* – 6 (Au: 5)	*Greater convenience* – 20.0	*Ease of contacting practitioner post-procedure* – 4.6	*Post-operative care* – 21.5
*Iraq* – 4	*‘Medical tourism’ in general; novel, drug-resistant infection and stem-cell therapy* – 3 each	*Lawyer* – 3	*State-of-the-art facilities* – 20.0		*Procedural risk* – 16.9
*Brazil* – 3	*Other* – 2	*Kidney donor* – 2 (F: 1; Au: 1)	*High-quality services* – 15.4		*Difficulty in contacting practitioner post-procedure* – 6.2
*Colombia and South Africa* – 2 each		*Politician* – 1 (Au: 1)	*Personalised service* – 15.4		
*‘Asia’ in general* – 1			*Surgeon/practitioner expertise* – 6.2		
*No country named* – 8			*Access to ‘medical breakthrough’* – 4.6		
			*Access to latest technology* – 3.1		

* Counts sum to more than 65, as some items cover more than one medical focus, or include more than one news actor.

** This refers to the percentage of stories to feature mention of the particular appeal, credibility or risk in question.

**Key and Definitions for Tables **2**and**3.

**F:** female.

Au: Australian (where ‘Au’ is not indicated, none of the news actors in that category were Australian).

News actors:

• Expert: specialists in medical and health-related disciplines, researchers/scientists in fields relevant to the story content, surgeons and representatives of medical-professional bodies.

• ‘Other’: individuals who were difficult to classify or who appeared in very small numbers; includes journalists/editors, social commentators and ethicists.

Coding categories for characteristics of media coverage on medical tourism (after Mason and Wright 2011).

Appeals: features of medical tourism mentioned in an item, either by the journalist or by a news actor, as attractive for a potential or actual patient.

• Ability to ‘feel good’: the opportunity for patients to feel better about their appearance or increase confidence in their looks as a result of the procedure.

• Access to services not available (for whatever reason – except cost) at home.

• Access to latest technology: the fact that a facility has the most modern medical equipment/techniques.

• No waiting time: the fact that patients going overseas for procedures can effectively ‘jump the (real or perceived) queue’.

• Greater convenience: the ability to have a procedure performed at the patient’s convenience rather than when facilities/doctors are available.

• High-quality services: patients leave satisfied with the outcome of their procedure(s).

• Longer hospital stays: the opportunity for greater hospital recuperation time.

• Low cost: the lesser cost of procedures, as compared to Australia/elsewhere.

• Medical breakthrough: the ability to access a very new treatment or procedure.

• Personalised services: how good care is/how well patients are looked after.

• Physician or surgeon expertise: patient confidence in their treating doctor’s experience and level of education.

• State-of-the-art facilities: the quality of accommodation or ‘extras’ offered by hospitals (such as meals and recreation facilities).

• Travel opportunity: the chance to have a holiday as well as surgery.

Credibility: dimensions of the medical tourism experience mentioned in an item, either by the journalist or by a news actor, to give it integrity or authority in the mind of a potential or actual patient.

• Accreditation: that the facility is independently accredited by some body of international standing.

• Ease of contacting practitioners post-procedure: medical practitioners making themselves easily available for follow-up in case of questions or complications.

• Logo-/branding symbol: inclusion of a facility or medical tourism agency’s logo.

• Physician or staff biography and education: mention of where medical practitioners were trained, or where they may previously have worked.

• Reference to number of international patients: mention of how many overseas patients (are believed to) visit a certain country or facility each year.

• Use of testimonies: inclusion of case-studies/profiles of satisfied patients.

Risks: aspects of the medical tourism experience mentioned, either by the journalist or by a news actor, as a source of actual or perceived risk (and perhaps as a reason not to proceed with an overseas medical procedure).

• Complications: the risk (or actuality) of complications as a result of a procedure.

• Ease of contacting practitioners post-procedure: the real or perceived concern that practitioners will not be easily available in case of questions or complications following a procedure.

• Ethical dilemmas: expressed concern that a procedure, or some dimension of it, is morally troubling.

• Exposure to novel risks: the potential for (or reality of) particular medical risks because of the location in which the procedure was performed.

• Legal recourse: the fear (or actuality) that due process may not be available in the case of anything going wrong as a result of an overseas medical procedure.

• Postoperative care: concern with regard to the standard or availability of post-operative care.

• Procedural risk: explicit mention of risk inherent in the procedure itself.

• Uncertainty as to what’s on offer: expressed concern as to quality of care, standards of overseas medical training, treatment or care, sterility in foreign medical facilities or the source of biological material (such as organs, eggs or sperm).

Related concepts within each of these categories are discussed, and sample characteristics for both television and newspaper items described in detail, in the next section. The first author coded the entirety of the dataset. A selection of 20% of the items, chosen by a random number generator from across the television and newspaper corpus, was then analysed by the second author.

## Results

Sixty-seven items of television news and current affairs coverage concerned with some aspect of international travel for medical treatment were identified, from a total of 28 580 items in the AHNRC’s database, of which 1355 were specifically about LMIC health. One item about medical tourism inbound to Australia was excluded. There were 90 potential newspaper items identified and then further checked to remove duplicates and assess relevance, as described in the previous section, leaving 65 items for analysis. Due to the numerous aspects of appeal, credibility and risk to be compared for each media item, agreement between authors was assessed by calculating the proportion of concepts on which both authors agreed. A high proportion of agreement was found for both television (81.6%) and newspaper (80.2%) items. For both Australian television and newspaper coverage, media interest in medical tourism peaked in 2007–08 (Table 1), with a further peak in newspaper coverage during 2010–11. News, as opposed to feature, stories focused on the subject were chiefly about the growth in ‘transplant tourism’, especially in relation to the sources of organs used and the ethics of their collection.

Television coverage of medical tourism was almost entirely focused on Asian countries (*n* = 63) (Table 2). The majority of medical concern in this media content was with cosmetic surgery (*n* = 20), stem-cell treatments (*n* = 15) and a variety of reproductive therapies (*n* = 13) including overseas surrogacy and gender-selective *in-vitro* fertilisation (IVF). Although there were several items about the controversial area of ‘transplant tourism’ (*n* = 9) more complex procedures and possible consequences, such as novel, drug-resistant infections that might be introduced by returning medical tourists (*n* = 2), were not well-represented overall in the dataset. Patients were the most common ‘news actors’ (individuals interviewed) (*n* = 69) to speak about medical tourism in television news and current affairs, over 80% of them female (*n* = 58). Among other news actors, ‘experts’ – medical specialists, researchers and scientists in relevant disciplines and representatives of the medical professions – featured heavily (*n* = 68), around half of them Australian. About three-quarters of the remaining expert commentators were from the LMIC contexts with which the television news items were concerned, the remainder being from other high-income nations. None of those involved directly in performing overseas procedures were Australian, but commentary from domestic professionals was often sought on, for example, the wisdom of travelling for medical treatment. Other stakeholders featured in television stories included medical tourism facilitators and representatives of overseas hospitals (*n* = 26), patients’ family members (*n* = 22), political actors in various locations (*n* = 8) and individuals from destination countries such as kidney donors (*n* = 13) and surrogate mothers (*n* = 2).

‘Access to services’ was most common among the attractions of medical tourism mentioned in the television dataset (referred to in 62.1% of stories). Subsequent appeals included low cost (36.4%), being able to ‘feel good’ (21.2%), the opportunity to travel (16.7%) and the lack of waiting time (13.6%). As to the characteristics of stories that gave credibility to medical tourism, personal testimonial was the most-used technique (referred to in 50% of stories), which is consistent with the large number of patient news actors. The number of international patients, or reference to an estimate of such figures, was also frequently cited to lend credence to the medical-tourism phenomenon or to a particular destination (42.4%). Finally, of the risks mentioned, ‘ethical dilemmas’ was the largest single category (referred to in 53% of stories). A sense of uncertainty about medical tourism – for instance, in relation to the quality of treatment, standard of practitioner qualification or sterility of equipment – also pervaded the television dataset (50%).

Without the same obligation as television to match textual content with constantly-changing images, newspaper stories (Table 3) were more wide-ranging in their interest; many items mentioned several countries or types of procedure rather than just a few examples. The broader geographical focus is evidence of this trend, although Asian nations still predominated (*n* = 59). Cosmetic surgery again dominated in relation to medical focus (*n* = 29), even more so than in the television coverage. The newspaper items were generally concerned with interventions of greater or lesser complexity, such as orthopaedic, dental and cardiac surgeries – but the second-largest single group of stories was about the contentious area of ‘transplant tourism’ (*n* = 22). There were also fewer types of news actors, although the pattern of those represented was similar to that in the television coverage: experts (*n* = 56), about 80% of them Australian, with the remainder split fairly evenly between individuals from LMICs and high-income nations; patients (*n* = 27), medical tourism facilitators and hospital representatives (*n* = 23), government spokespeople or politicians (*n* = 16) and patients’ family members (*n* = 7).

The tone of the newspaper dataset was much more a marketing one, with the main appeal being that of low cost (referred to in 53.8% of stories), with travel opportunities (32.2%) and ability to ‘feel good’ (29.2%) also important. However, access to services (36.9%) and lack of waiting time (33.8%) emerged strongly because of the number of stories about ‘transplant tourism’. The newspaper items contained fewer personal testimonials than did the television data (18.5%). Instead, their major means of establishing credibility was via reference to the number of international patients visiting a country or facility for medical-tourism purposes (35.4%). In addition, the emphasis in any mention of risk was foremost about the procedures themselves – uncertainty (69.2%), possible complications (47.7%) – and only then about the ethical dimensions of the practice (36.9%). This approach, and a certain perception of LMICs, was perhaps best summed up in one television story when an Australian provider of domestic cosmetic surgery asked rhetorically during an interview, ‘if you can’t drink the water there, why would you let them operate on you?’.

## Discussion

This study examined Australian television and print news and current affairs coverage of medical tourism: its type and format, content – the countries, types of procedures and news actors featured – and the extent to which the appeals, credibility and risks of medical tourism were mentioned. This section considers what messages about medical tourism and its LMIC destination countries were presented in the coverage.

The media portrayal of medical tourism reflects several trends identified in earlier research concerning the Australian domestic coverage of both LMICs and their health status [8]. First, the topics represented among the 131 media items analysed were concentrated around a total of just ten major medical foci (Tables 2 and 3): a range of surgical interventions, reproductive and regenerative procedures, and the threat of novel infections brought into the country by returning medical tourists. This set of concerns is similarly narrow to those previously noted in an investigation of the Australian media’s reporting of international humanitarian issues [4]. Geographic attention in both television and newspaper items was largely on Asian nations, due to their proximity and consequent significance as a cluster of inexpensive destinations with which Australians already have some familiarity as both ‘backyards’ and ‘playgrounds’ [36]. The newspaper data evidenced somewhat more extensive geographic and medical emphases. Yet this broader focus did not extend to risk considerations, which remained largely limited to individual patients’ personal or legal interests. This latter observation reflects the findings of a Canadian qualitative study of medical tourists, who spoke about the ethical dimensions of their particular decision to travel for treatment in terms of what they perceived as aspects of domestic health provision that had forced them abroad: namely, the waiting times and systemic limitations which, in turn, justified their ‘queue-jumping’ [37].

Second, the restricted medical, geographic and risk concerns evident in the Australian media coverage of medical tourism were reinforced by its emphasis on identified individuals who had undergone surgery. That patients featured so prominently among news actors in both television and newspaper coverage is consistent with the use of sources in health and medical news: those affected by a health problem provide an appealing and ‘authentic’ contrast to the media presentation of statistics or research [30]. Yoking such ‘newsworthy’ but otherwise abstract material to an individual narrative personalises the story, in line with the centrality of ‘human interest’ to general news and current affairs [38]; the items in these datasets invariably used medical tourists’ experiences as ‘hooks’ for a wider discussion of the phenomenon. Although not all patient news actors had happy experiences to relate, every story that presented medical tourism in a positive light included at least one delighted patient. Third, the high proportion of Australians among all those interviewed mirrors the inclination toward domestic sources in LMIC news more broadly [8]. There was far less media attention given to those who make certain types of medical tourism possible, such as surrogate mothers and organ donors.

Finally the extent to which the media content sought to establish a sense of personal relevance for audience members, a characteristic that has previously been noted in the Australian coverage of LMIC health [8], partially explains the patterns of appeals, credibility and risks in the presentation of medical tourism. Among the television items, the attraction of ‘access to services’ appeared most frequently as a result of the number of stories about stem-cell and reproductive therapies not legally available to patients in Australia, with ‘access to ‘medical breakthrough” not much further down the list (referred to in 21.2% of stories). The focus on these procedures, too, made ‘ethical dilemmas’ (53%) the largest single category of risk evident in the television coverage. Subsequently in both television and newspaper datasets the common appeals of low cost, being able to ‘feel good’, the opportunity to travel and the lack of waiting time were consistent with the large amount of coverage related to cosmetic surgery, which was presented as a matter of ‘lifestyle choice’ for those willing and able to pay. Among the newspaper items nearly half mentioned the risk of complications (47.7%), as a result of the interest in certain, more complex (transplant and orthopaedic) surgeries. Portraying medical tourism as an extension of the bargain-hunters’ holiday that Australian travellers in Asia have long enjoyed, on which the greatest satisfaction is derived in purchasing desirable goods at the lowest possible price, promotes a kind of medical ‘shop-til-you-drop’ approach, with unrestricted access to procedures that are not necessarily required or recommended – and ultimately, a commodification of health-care [24].

Given the various dimensions of uncertainty surrounding medical tourism, we might assume that potential medical tourists approach this healthcare option with heightened perceptions of its associated risks [35]. Yet in its presentation of medical tourism, Australian news and current affairs coverage of the practice more often referenced some aspect of the actions of other medical tourists (the numbers who take part, and their personal experiences) than any reliable medical consideration. Mentions of a health facility’s international accreditation (referred to in 12.1% and 4.6% of television and newspaper stories, respectively), medical practitioners’ biography or education (10.6% and 12.3%) and ease of contacting a health-care provider following a procedure (6% and 4.6%) ranked fairly low down the list of such factors in both television and newspaper items. There is little opportunity for individuals to verify this key information and, at any rate, few medical tourists would have the requisite knowledge to properly assess a hospital’s reputation or a doctor’s skills for themselves – despite the confident assertion by many patient news actors that they had ‘done their research’ online before committing to travel. An interview study with Canadian medical tourism facilitators found that most of their ‘referrals’ came via word-of-mouth or websites [39] – and crucial sources of relevant online information are offered by commercial interests [40]. Investigations into the presentation of appeal and risk on medical tourism websites have previously noted that testimonials, a common technique in general advertising and used liberally in this Australian media dataset, are of limited value to would-be medical tourists since they provide no insight into the individual-level differences that might influence medical outcomes [35].

Such a presentation is troubling since the notion of ‘choice’ and the associated power of the healthcare consumer are central to the medical tourism phenomenon [16] and feature prominently in its Australian television and newspaper coverage. The mention of diverse and contrasting appeals and risks across the media dataset would appear to reinforce a belief that audiences, as an exercise of their freedom to choose, can make up their own minds. This approach is also understandable in editorial terms, with ‘balance’ a significant tenet of journalistic practice. However, presenting information from sources of varying legitimacy as though they were equally valid might properly be considered a form of bias [41] and may leave audience members confused as to their best course of action. The television items examined here appeared largely on commercial networks, which are under sustained pressure to produce widely-engaging content at the lowest cost [42]. In this context feature stories, which comprised the bulk of this coverage (Table 1) and that reported medical tourism as a minority practice in Australian social life, make both economic and ratings sense [8]. That the print items were mostly published in metropolitan newspapers reflects the mainly urban distribution of Australia’s population. It also suggests that this coverage does not merely give an account of the current domestic reality of medical tourism but is also aspirational, demonstrating to a wide and relatively affluent audience why and how they might participate in the practice.

Since our findings showed that both television and newspaper portrayals placed greater emphasis on the appeals than the risks or factors lending credibility to medical tourism, it was perhaps unsurprising that the ethical interest expressed in this coverage was also largely at the level of the individual Australian patient, their experiences and feelings about the process. Canadian research into medical tourists’ own understanding of their health-related travel has demonstrated a disjunction between the system-level ethical concerns of academic literature on the practice and the personal ones expressed by medical tourists; indeed, many of those interviewed were puzzled by questions about any possible larger ethical implications [37]. Yet as mentioned above medical tourism has huge, potential medical and political consequences for both source and destination countries. While it doubtless benefits some patients from high-income nations and the large corporate medical outfits that have increasingly arisen to serve this market [24], the advantages for local populations – including ‘direct’ providers like surrogate mothers and organ donors – are less certain [16,26]. In our data one, lengthy television current affairs story and three shorter follow-up pieces examined the gap in quality between the private healthcare offered to medical tourists in India and the public services available to that country’s citizens, but these were the only media items to engage with the possible effects of medical tourism for health in LMICs. Four stories – one on an overseas knee reconstruction and three about cosmetic surgery – mentioned some health-system outcomes, but only insofar as they related to subsequent burdens for Australian healthcare.

Presenting medical tourism as simply another option available to the wealthy may inhibit appropriate policy development in source countries as, for example, growing numbers of medical tourists diminish the incentives for governments to expand their domestic health workforces [43]. Although in recent years private organisations such as the US-based Joint Commission International (JCI) have accredited health-care facilities in numerous LMICs [44], medical tourism otherwise remains largely unregulated: Australia and Canada, for instance, have no national health and safety guidelines on patient or practitioner involvement in the practice [45]. Likewise efforts in destination countries have, to date, been piecemeal: India now has a special medical tourist visa but has otherwise left sectoral regulation to its private medical providers [46]. Many medical-tourism destinations have less strict medical liability provisions than source countries, restricting patient options for legal recourse and compensation; some medical tourism facilitators include insurance in their prices and patients may take out their own policies [10,14]. In the absence of official, medical directives and within the prevailing framework of medical tourism as a customer’s prerogative, the presentation to Australian media audiences of any hazards arising from the practice was a combination of anecdotal, patient evidence and a healthy dose of ‘buyer beware’.

Equally instructive in examining the content of any media corpus is the matter of what it does *not* contain. Cosmetic surgery was, until recent times, reasonably uncommon and presented to media audiences as mainly the province of professionally vain female celebrities, whose medical outcomes were sometimes the occasion for a mixture of bemusement and horror [47]. This cultural dynamic has clearly shifted. Across the television and newspaper items investigated here, cosmetic surgery was the dominant medical focus, yet never once were the – again, mainly female – patients censured for vanity. Instead their decision to do ‘something that I’ve always dreamed about’ and fix ‘a few imperfections’ was portrayed sympathetically, and as largely another manifestation of consumer choice – in this case an economically rational one, since the decision to go overseas was so often presented as being motivated by the lower prices charged for such procedures elsewhere. It is also interesting to consider how medical tourism would be presented in the domestic media if the phenomenon looked similar to its LMIC manifestation: namely, small but growing numbers of wealthy overseas patients travelling to Australia for health-care. A recent scoping study, prepared for the Australian government, on inbound medical tourism gives some idea of the perceived benefits from this practice. Again, they are presented in highly rational, mostly economic, terms: attracting foreign currency, reducing the medical professional ‘brain drain’ of health workers and providing extra resources for investment into the local health system [48]. The study points out that Australian education is already marketed to international student ‘customers’ in the same way that medical services now might be.

The context for most of the world’s travel for medical care is quite banal: it would appear to take place largely between LMICs themselves, over short distances, across borders and within regions, although there is a lack of valid data on the size and direction of such patient flows [14,46]. However, media coverage of the practice for Australian audience presented it as being primarily about long-distance journeys for non-essential, often cosmetic, procedures. The picture offered in this television and newspaper data of LMICs themselves was similarly distorted: no longer simply passive recipients of external financial and technical assistance these nations were now sources of benefit to Australians, in the form of low-cost, convenient and even enjoyable combinations of health-care and travel. In this, the Australian media’s presentation of medical tourism departs from how LMICs are usually covered in mainstream news and current affairs. Rather than attracting attention because of the health problems felt to be ‘typical’ of such locations – communicable disease, injury and child health, with no emphasis on emerging problems such as chronic disease [8] – instead it is LMICs’ credentialled experts and advanced facilities that are touted to local audiences. The ambivalence and complexity of LMIC destinations courting medical tourists in national self-interest while, to varying degrees, failing to adequately meet the health-care needs of their own citizens [23] is a poor fit with the simpler Australian media narrative of individual choice and personal gain. Medical tourism is likely to continue growing, with increased foreign investment in private health-care in LMICs, improved access to technology in these countries, continued ‘word of mouth’ about the practice, the intensification of its marketing and persistent cost differentials between source and destination countries [13]. In addition, many American insurers are moving toward sending patients requiring complex medical procedures offshore in their attempts to reduce the financial burden of employee healthcare [19]. This growth is significant because, although medical tourism has consequences for both social justice and health equity, what it will mean in the longer term for public health is far from settled.

There are several limitations to the current study. Although there was careful and comprehensive quantification of the content categories discussed, this coding could not account for the quality, importance or strength of each of these elements within the television or newspaper items surveyed. Further, this research could not account for any effects on potential medical tourists’ decision-making of the media content examined. Future studies into the media coverage of medical tourism could usefully address each of these areas by continuing qualitative research with past or potential medical tourists [15] in order to better understand how elements of appeal, credibility and risk played a part in their choice; and undertaking comparative analysis of similar media datasets from other destination and source countries.

## Conclusions

The present research explored the content of Australian television and newspaper coverage of medical tourism, and the presentation of both medical tourism and its LMIC destinations. It revealed that this portrayal is in line with broader domestic media coverage of LMIC health, with its narrow medical, geographic and ethical foci, and emphases on Australian participants and commentators as the principal actors through whom the medical tourism phenomenon is understood. In addition the impression of medical tourism advanced to audiences is a quite specific one, of affluent customers for health-care making rational choices based on individual desire for particular services (low cost, ability to travel and being able to ‘feel good’) and appetite for risk (uncertainty). Within this consumer-focused frame, the patient experience and medical outcome are presented as being of equal importance, and any broader concerns are pushed aside. As medical tourism to LMICs is increasingly perceived as a viable health-care option for citizens of nations such as Australia, understanding its appeals to audiences will become more important.

## Endnotes

^a^ Countries that feature in news items used in preparing this paper are all identified as low- or middle-income countries, as defined by WHO [49].

## Abbreviations

AHNRC: Australian Health News Research Collaboration; IVF: *In-vitro* fertilisation; JCI: Joint Commission International; LMIC: Low- and middle-income country.

## Competing interests

The authors declare that they have no competing interests.

## Authors’ contributions

MI designed the study, conducted the initial data analysis and coding, and calculated the proportion of agreement. MI and SS reviewed the coding, developed the content categories and wrote the paper. Both authors read and approved the final manuscript.

## Pre-publication history

The pre-publication history for this paper can be accessed here:

http://www.biomedcentral.com/1471-2458/13/109/prepub
